# CRISPR/Cas13-assisted hepatitis B virus covalently closed circular DNA detection

**DOI:** 10.1007/s12072-022-10311-0

**Published:** 2022-03-17

**Authors:** Xiangying Zhang, Yuan Tian, Ling Xu, Zihao Fan, Yaling Cao, Yingmin Ma, Hao Li, Feng Ren

**Affiliations:** 1 Beijing Youan Hospital, Capital Medical University, No. 8, XitouTiao Road, Youwai Street, Fengtai District, Beijing, 100069 China; 2 State Key Laboratory of Pathogen and Biosecurity, Beijing Institute of Microbiology and Epidemiology, Beijing, 100071 China

**Keywords:** Hepatitis B virus, Covalently closed circular DNA, HBV cccDNA detection, CRISPR/Cas13, Collateral effect, Droplet digital PCR, Quantitative PCR, Rolling circle amplification, Southern blot, Antiviral therapy

## Abstract

**Background and aims:**

The formation of an intranuclear pool of covalently closed circular DNA (cccDNA) in the liver is the main cause of persistent hepatitis B virus (HBV) infection. Here, we established highly sensitive and specific methods to detect cccDNA based on CRISPR-Cas13a technology.

**Methods:**

We used plasmid-safe ATP-dependent DNase (PSAD) enzymes and HindIII to digest loose circle rcDNA and double-stranded linear DNA, amplify specific HBV cccDNA fragments by rolling circle amplification (RCA) and PCR, and detect the target gene using CRISPR-Cas13a technology. The CRISPR-Cas13a-based assay for the detection of cccDNA was further clinically validated using HBV-related liver tissues, plasma, whole blood and peripheral blood mononuclear cells (PBMCs).

**Results:**

Based on the sample pretreatment step, the amplification step and the detection step, we established a new CRISPR-Cas13a-based assay for the detection of cccDNA. After the amplification of RCA and PCR, 1 copy/μl HBV cccDNA could be detected by CRISPR/Cas13-assisted fluorescence readout. We used ddPCR, qPCR, RCA-qPCR, PCR-CRISPR and RCA-PCR-CRISPR methods to detect 20, 4, 18, 14 and 29 positive samples in liver tissue samples from 40 HBV-related patients, respectively. HBV cccDNA was almost completely undetected in the 20 blood samples of HBV patients (including plasma, whole blood and PBMCs) by the above 5 methods.

**Conclusions:**

We developed a novel CRISPR-based assay for the highly sensitive and specific detection of HBV cccDNA, presenting a promising alternative for accurate detection of HBV infection, antiviral therapy evaluation and treatment guidance.

**Supplementary Information:**

The online version contains supplementary material available at 10.1007/s12072-022-10311-0.

## Introduction

Hepatitis B virus (HBV) infection continues to be a major health burden worldwide. The global HBV chronic infection is approximately 257 million people [1]. Eliminating HBV infection has become an important task for governments worldwide. In 2016, the Global Health Sector Strategy on Viral Hepatitis was approved by the World Health Assembly to eliminate HBV infection by 2030 [2].

In clinical treatment, functional cure of HBV infection refers to the disappearance of sustained HBsAg with or without the presence of anti-HBs, while complete cure is defined as eradication of HBV covalently closed circular DNA (HBV cccDNA) [3, 4]. Viral persistence as HBV cccDNA is a key obstacle to the cure of chronic hepatitis B (CHB) [5]. The key factor in the difficulty of curing CHB is the existence of HBV cccDNA, which is a template for the replication of HBV RNA and virus-derived progeny because it can be continuously and stably stored in the hepatocyte nucleus and cannot be eliminated by any antiviral drugs. Therefore, it is necessary to detect the persistence of HBV cccDNA, which contributes to guiding further clinical therapeutics.

Despite the critical role of cccDNA in the chronicity and duration of HBV infection, there is a lack of effective and accurate detection methods to monitor cccDNA. There are two characteristics that limit the detection of HBV cccDNA [6]. The first obstacle to cccDNA detection is that cccDNA levels are extremely low copy number, averaging 0.1 to 1 copy per hepatocyte [7], requiring a highly sensitive detection method; the second one is how to distinguish cccDNA from relaxed circular DNA (rcDNA), whose sequences are highly homologous to cccDNA sequences; thus, detection methods must also have high specificity. Therefore, a highly sensitive and highly specific HBV cccDNA detection method is extremely important to further explore therapeutic drugs targeting cccDNA formation and maintenance. The widely accepted method for cccDNA detection is the Southern blot, which can effectively distinguish cccDNA and rcDNA based on differences in electrophoretic mobility [8]. But it is insensitive, complex, time-consuming and not suitable for high-throughput drug screening. At present, many new methodologies, including polymerase chain reaction (PCR)-based methods, have recently been applied to detect and quantify cccDNA. For example, quantitative PCR (qPCR), which amplifies across the gaps present in rcDNA, can achieve some specificity for cccDNA but is still required to reduce the false-positive amplification of rcDNA [9, 10]. Rolling circle amplification (RCA) is a potential alternative to detect cccDNA by increasing the sensitivity, but it is not widely accepted [11]. Digital PCR is an effective method for the detection of HBV cccDNA. It has good sensitivity and specificity, but it is expensive and requires special equipment; therefore, digital PCR cannot be widely used [12, 13]. Therefore, a new method for detecting HBV cccDNA with high sensitivity and specificity needs to be further explored.

The clustered regularly interspaced short palindromic repeats (CRISPR)-associated protein (Cas) system is an acquired immune system for the cleavage of foreign genetic elements from invading viruses and phages and was first identified in bacteria and archaea [14]. Importantly, upon binding to target double-stranded DNA (dsDNA) or RNA, several Cas proteins, such as Cas12,13,14, can be activated to perform the nonspecific degradation of nontargets (trans-cleavage) after the specific recognition of nucleic acids, thus providing a novel diagnostic approach for nucleic acid detection [15, 16]. The CRISPR-Cas system combined with nucleic acid amplification could rapidly detect DNA or RNA viruses with high sensitivity as low as 50 fM [17]. These advantages of the CRISPR-Cas detection system can compensate for the shortcomings of the current HBV cccDNA testing methods.

Here, we established a high-sensitivity and high-specificity HBV cccDNA detection method using the CRISPR-Cas system combined with RCA and PCR methods. For a more comprehensive evaluation, we collected clinical samples that were detected by quantitative real-time PCR (qPCR), droplet digital PCR (ddPCR) and our CRISPR-based cccDNA assay.

## Methods

### Patients and sample collection

Liver samples were collected from 24 patients with HBV-related Hepatocellular carcinoma (HCC), 6 patients with HBV-related Liver cancer (LC), 10 patients with CHB and 3 individuals with normal livers. Normal liver specimens were collected from hepatic resection for liver transplantation. CHB samples were obtained from patients undergoing liver puncture biopsy. LC and HCC liver samples were obtained from the livers of patients with HBV infection undergoing liver transplantation. Whole blood, plasma, and peripheral blood mononuclear cells (PBMCs) were collected from 24 patients with HBV affected by different HBV viral loads and 3 healthy subjects. The study was approved by the medical ethics committee of Beijing Youan Hospital, Capital Medical University, and written informed consent was obtained from each patient. The procedures followed were in accordance with the ethical standards of the responsible committee on human experimentation and with the Helsinki declaration of 1975, as revised in 1983.

### Statistical analysis

The HBV cccDNA results are expressed as copies/µL (template concentration). Means and standard deviations were calculated by GraphPad Prism software version 8.0 (GraphPad, Inc., La Jolla, CA, USA). Mean differences in quantification were determined by paired *t*-test and Fisher’s exact test. All statistical tests were two-sided, and *p* < 0.05 was considered statistically significant.

Other methods in supplementary materials.

## Results

### Schematic of HBV cccDNA using a CRISPR-based cccDNA assay

**Fig. 1:**
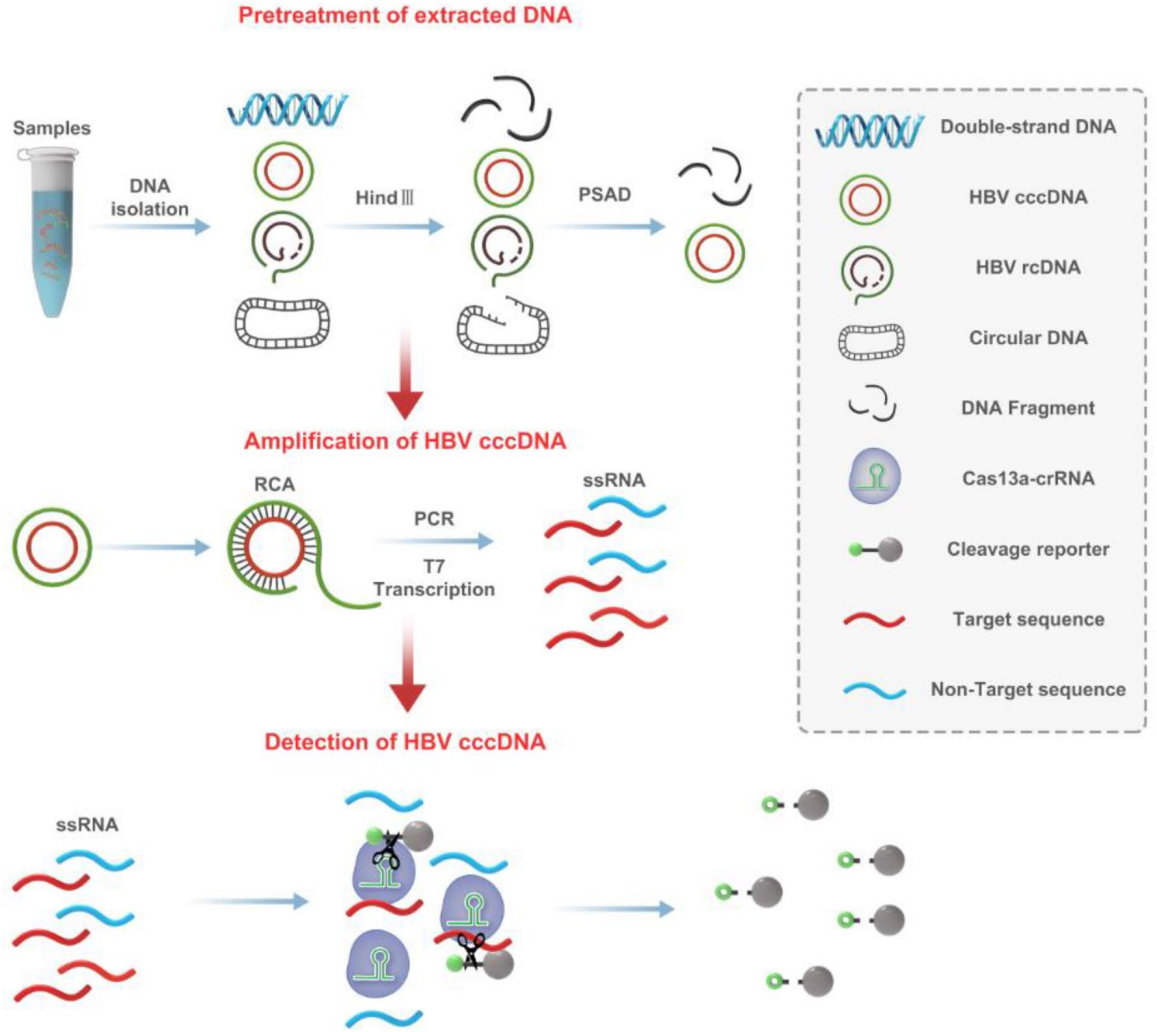
Design and working principle of the CRISPR-based cccDNA assay for HBV cccDNA detection. Total DNA was extracted from samples and pretreated with HindIII and PSAD. Amplification of HBV cccDNA. The pretreated samples were amplified by RCA and PCR, which transcribed the target sequence into single-stranded RNA. To detect HBV cccDNA, specific crRNA directs Cas13a to recognize target RNA, triggering collateral activity of Cas13a to cleave RNA reporters, which can be visualized by fluorescence signal

In this study, we established a novel strategy named the CRISPR-based cccDNA assay for the highly sensitive and specific detection of HBV cccDNA. As illustrated in Fig. 1, the CRISPR-based cccDNA assay consists of a sample pretreatment step, an amplification step and a detection step. In the sample pretreatment step, total DNA was pretreated with HindIII and PSAD to increase the sensitivity and specificity of cccDNA detection. In the amplification step, the pretreated samples were used as templates for RCA amplification, and PCR amplification was performed to increase the amount of the target sequence that was transcribed into single-stranded RNA (ssRNA) using T7 RNA polymerase because the T7 promoter sequence was appended ahead of the PCR products, which made PCR products transcribe into ssRNA detected by Cas13a with a specific target crRNA. In the detection step, specific crRNA directs Cas13a to recognize target RNA that is complementary to the spacer of crRNA, thereby triggering Cas13a-mediated collateral cleavage of a reporter RNA, allowing for real-time detection of the target.

### The effect of Hind III and PSAD on the elimination of noncircular DNA

**Fig. 2:**
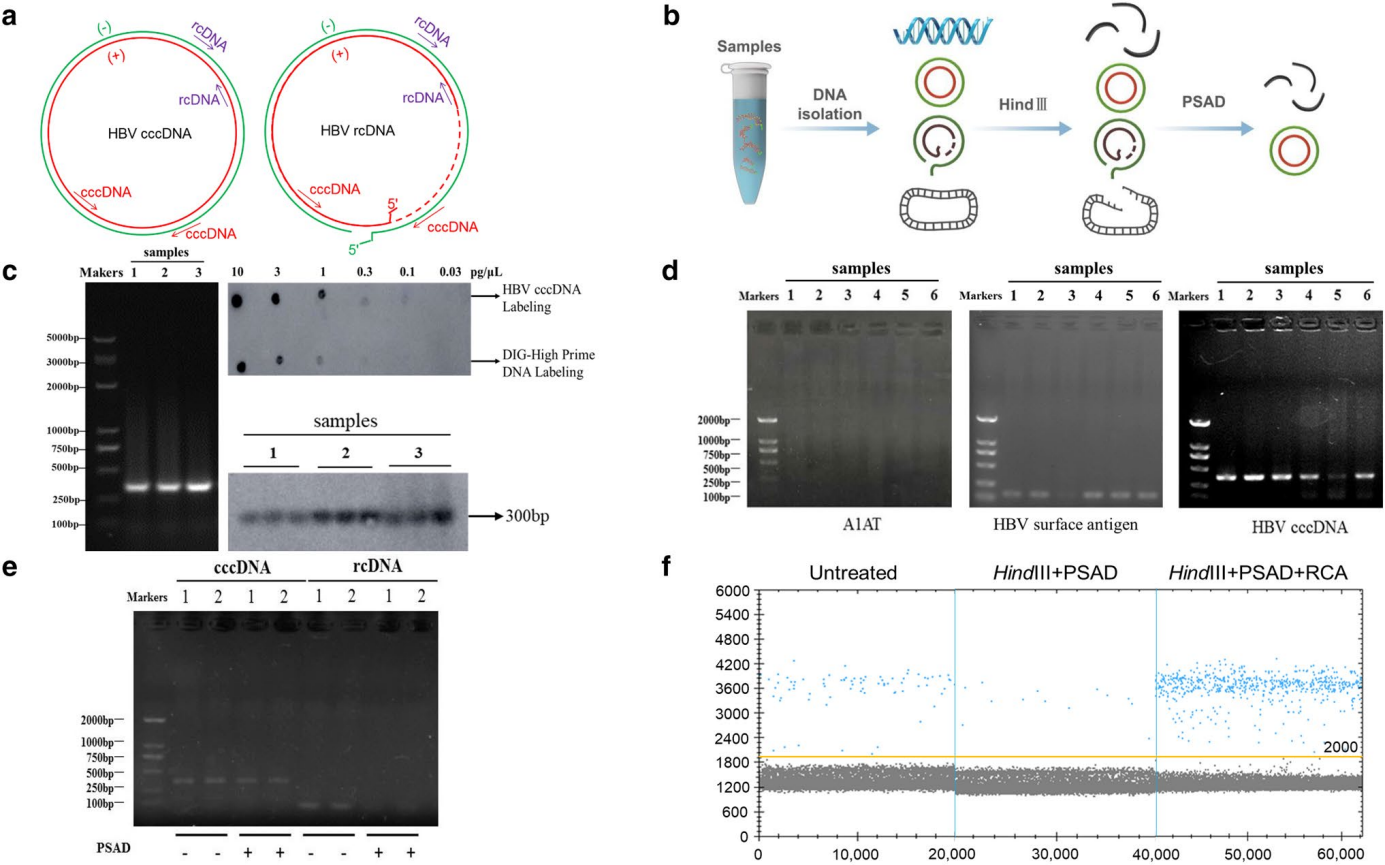
The effect of Hind III and PSAD on the elimination of noncircular DNA. **a** Schematic diagram of the structure of HBV cccDNA and HBV DNA. **b** Schematic diagram of samples pretreated with HindIII and PSAD. **c** Total DNA of three HBsAg-positive samples was extracted, amplified by PCR, and detected by agarose gel electrophoresis. Probe labeling was performed by HBV cccDNA and DIG-High-prime DNA to detect the HBV cccDNA of samples in nitrocellulose filter membranes. Three replicates were performed for each sample. **d** Total DNA after Hind III and PSAD digestion was amplified using primers for A1AT, HBV surface antigen and HBV cccDNA, and the products were detected by agarose gel electrophoresis. The length of PCR products of A1AT gene, HBV surface antigen gene and HBV cccDNA are 1224, 150 and 364 bp, respectively. **e** Total DNA after Hind III and PSAD digestion was amplified using HBV cccDNA and HBV rcDNA primers, and the products were detected by agarose gel electrophoresis. **f** The ddPCR assay was performed to detect the total DNA of an HBsAg-positive sample treated or untreated with HindIII and PSAD digestion, and the product after HindIII and PSAD digestion was amplified by RCA

According to the structural differences between rcDNA and cccDNA of HBV (Fig. 2a), the primers were designed at the location gap coding region of HBV rcDNA to specifically amplify the HBV cccDNA gene sequence. The schematic diagram showed that total DNA extracted from samples was digested with *Hind*III and PSAD (Fig. 2b). First, to confirm the presence of HBV cccDNA, three HBV surface antigen (HBsAg)-positive samples were treated with the abovementioned processes, and the products were detected by agarose gel electrophoresis (Fig. 2c). HBV cccDNA and DIG-High prime DNA labeling were performed, and products of three HBsAg-positive samples were detected by southern blotting to verify the existence of HBV cccDNA (Fig. 2c). Then, PCR amplification of six total DNA samples after Hind III and PSAD digestion showed that the A1AT gene, which is a glycoprotein widely present in liver tissues, was not detected by the agarose gel electrophoresis assay again, but the HBsAg gene can be detected, which comes from the HBV cccDNA, and products were dramatically detected after amplification of the HBV cccDNA primers in agarose gel electrophoresis (Fig. 2d). Additionally, the HBV rcDNA of the two samples was significantly decreased after digestion of *Hind*III and PSAD, but their HBV cccDNA remained basically unchanged (Fig. 2e). Furthermore, used the ddPCR assay to compare the number of copies of HBV cccDNA detected in three replicates of an HBsAg-positive sample treated or untreated with HindIII and PSAD digestion, which showed 48 ± 5 copies/μL and 255 ± 12 copies/μL, respectively (Fig. 2f). The product of HindIII and PSAD digestion was amplified by RCA, and three replicates (1974 ± 36 copies/μL) were tested in the same ddPCR experiment (Fig. 2f). Thus, these results demonstrated that the combined use of HindIII and PSAD digestion may effectively eliminate linear DNA molecules and HBV rcDNA to increase the specificity of cccDNA detection, and RCA markedly increases the number of copies of cccDNA.

### crRNA design and identification for the detection of HBV cccDNA

**Fig. 3:**
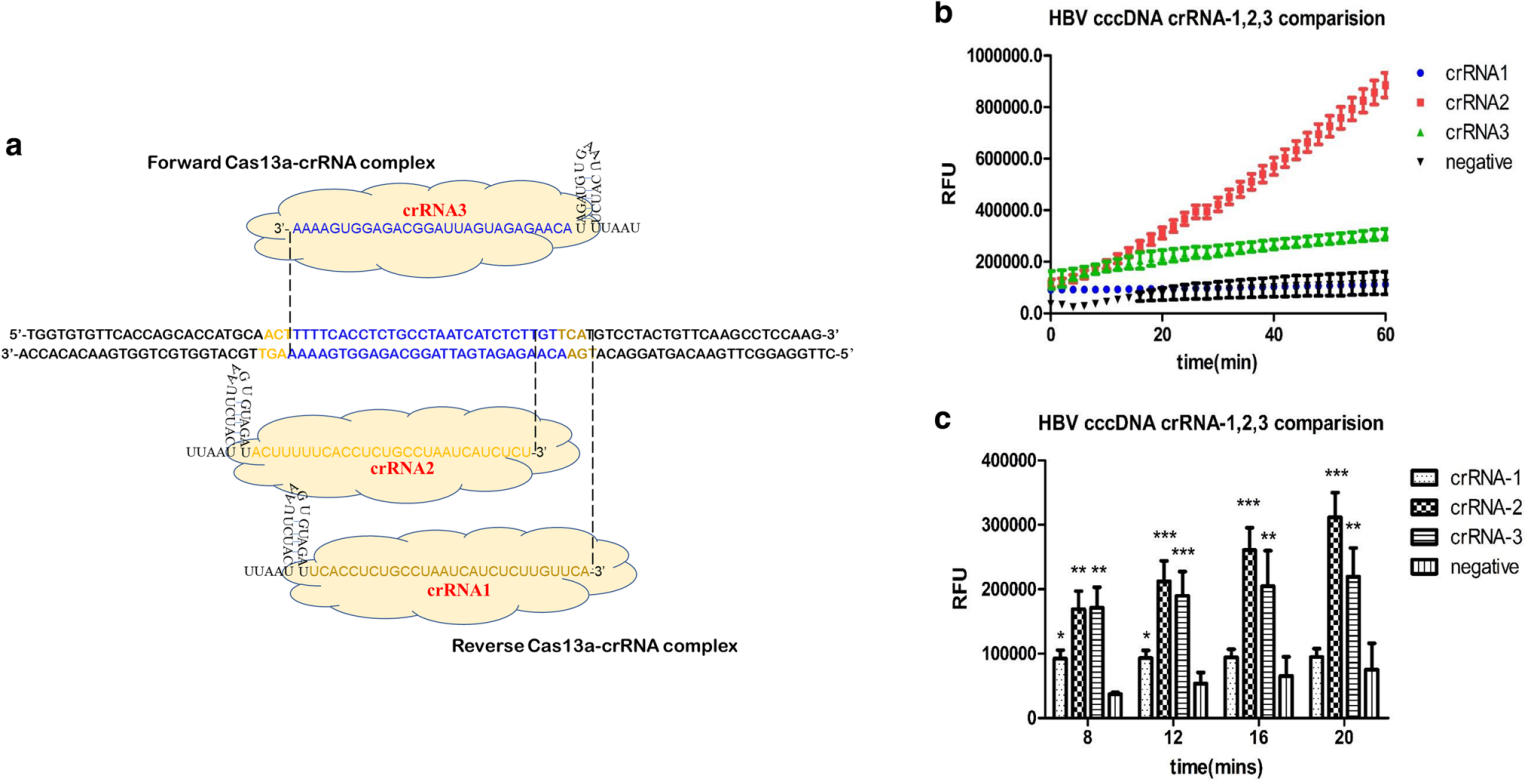
crRNA design and identification for the detection of HBV cccDNA. **a** HBV cccDNA genome map with the detailed sequence information of the designed crRNAs targeting the conserved sequence. For comparison purposes, crRNA1, crRNA2 and crRNA3 were designed at different sites. **b** Screening of crRNAs for HBV cccDNA detection by Cas13a collateral detection. **c** Fluorescence signals could be detected with all three crRNAs in different min through Cas13a-based reactions. Error bars indicate 1 S.D. based on three technical replicates; *p < 0.05, **p < 0.01, ***p < 0.001, compared with the negative group

To obtain efficient and specific crRNAs applicable for detecting HBV cccDNA using CRISPR/Cas13a, we designed three different crRNAs (crRNA1, crRNA2, and crRNA3) that were specific to different sites in the same HBV cccDNA amplification region (Fig. 3a). After RCA and PCR amplification of HBV cccDNA-positive samples and the use of a fluorescence detector by Cas13a, we verified the availability of three candidate crRNAs. As illustrated in Fig. 3b, the fluorescence signals of HBV cccDNA crRNA-2 (870,000.0 ± 70,000 relative fluorescence units (RFU)) and HBV-cccDNA crRNA-3 (250,000.0 ± 45,000 RFU) were markedly elevated compared with the negative control (140,000 ± 64,000 RFU) at 60 min, while HBV cccDNA crRNA-2 demonstrated a higher fluorescence signal than the other two crRNAs. Furthermore, we found that fluorescence signals could be detected for all three different crRNAs 8 min after Cas13a-based reactions, and HBV cccDNA crRNA-2 showed a higher fluorescence signal at 16 min than the negative control (280,000 ± 20,000 RFU vs. 50,000 ± 10,000 RFU, *p* < 0.001) (Fig. 3c). Thus, Cas13a is capable of more rapid detection of HBV cccDNA with HBV cccDNA-2 crRNA, so we selected HBV cccDNA-2 crRNA for the following study.

### Comparison of qPCR, PCR-CRISPR and ddPCR methods using HBV cccDNA standard

**Fig. 4:**
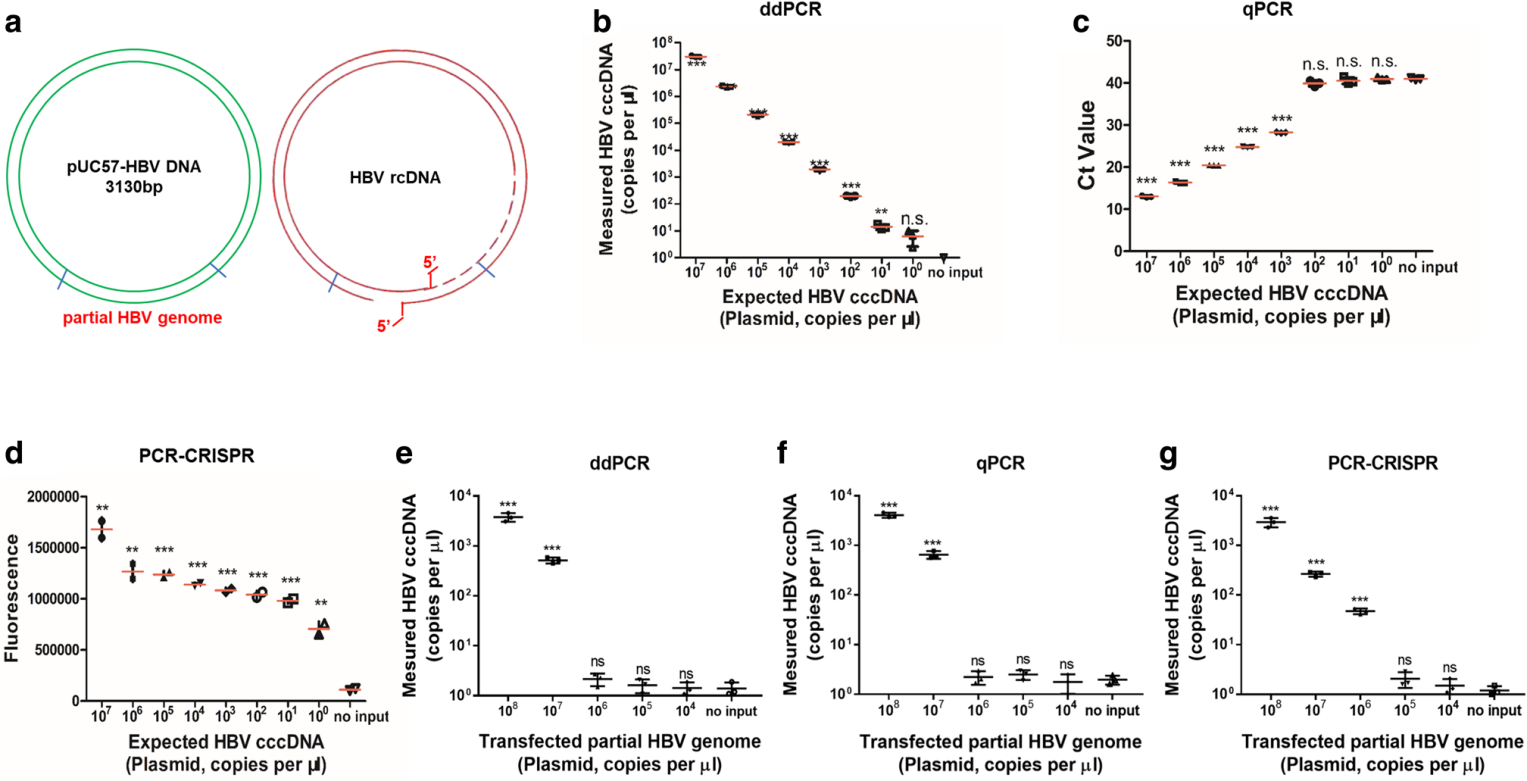
Comparison of qPCR, PCR-CRISPR and ddPCR methods using the HBV cccDNA standard. **a** Schematic diagram of HBV DNA plasmid structure. **b** Serial dilutions of the HBV cccDNA standard were detected by the ddPCR method. **c** Serial dilutions of the HBV cccDNA standard were detected by qPCR. **d** Serial dilutions of the HBV cccDNA standard were detected by the PCR-CRISPR method. **e** Serial dilutions of the HBV cccDNA in Huh7 cells were detected by the ddPCR method. **f** Serial dilutions of the HBV cccDNA in Huh7 cells were detected by qPCR. **g** Serial dilutions of the HBV cccDNA in Huh7 cells were detected by the PCR-CRISPR method. Error bars indicate 1 S.D. based on three technical replicates; *p < 0.05, **p < 0.01, ***p < 0.001, compared with the no-input group

Next, we explored the sensitivity of combining Cas13a-based detection with PCR amplification steps compared with qPCR and ddPCR methods. A schematic diagram of the HBV cccDNA plasmid sequence is shown in Fig. 4a. Using serial dilutions of the HBV cccDNA standard, we found that the sensitivity of the PCR-CRISPR method for HBV cccDNA was 1 copy/μL, which was higher than that of ddPCR detection approaches, whereas the qPCR method did not detect a sensitivity lower than 10^3^ copies/μL of target for the detection of the HBV cccDNA standard (Fig. 4b–d). Additionally, same experiments have been performed to detect Huh7 cells transfected with serial dilutions of HBV cccDNA plasmids. The results showed that the sensitivity of the ddPCR, qPCR and PCR-CRISPR method for HBV cccDNA were 10^2^ copies/μL, 10^3^ copies/μL and 10 copies/μL, respectively (Fig. 4e–g). Therefore, the Cas13a-based detection method further increased the sensitivity of the detection of the HBV cccDNA standard.

### Comparison of the qPCR, PCR-CRISPR, RCA-qPCR, RCA-PCR-CRISPR, ddPCR and RCA-ddPCR methods using positive liver samples of HBV cccDNA

**Fig. 5:**
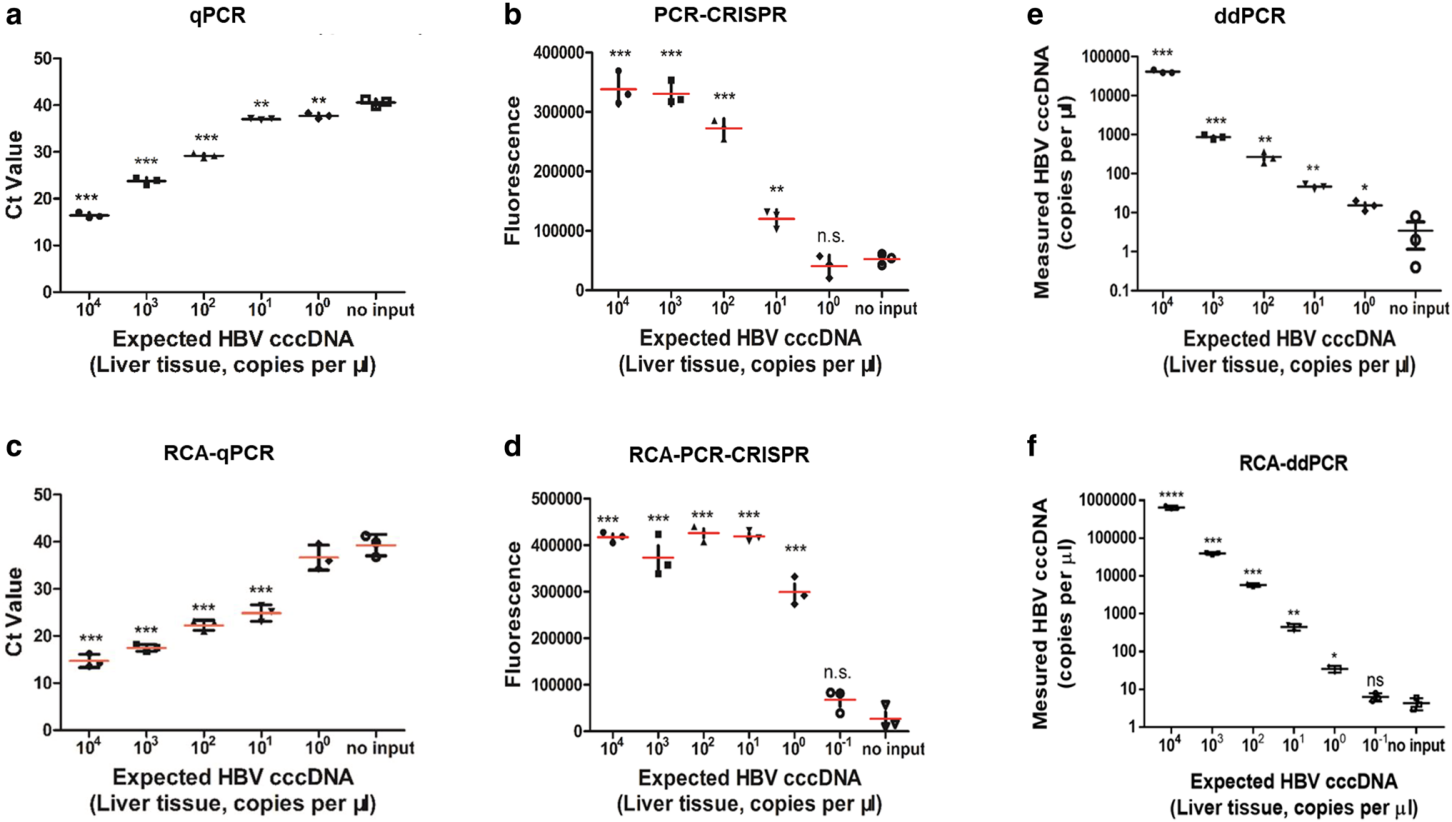
Comparison of qPCR, PCR-CRISPR, RCA-qPCR, RCA-PCR-CRISPR and ddPCR methods using positive liver samples of HBV cccDNA. **a** Serial dilutions of positive liver samples of HBV cccDNA were detected by qPCR. **b** Serial dilutions of positive liver samples of HBV cccDNA were detected by the PCR-CRISPR method. **c** Serial dilutions of positive liver samples of HBV cccDNA were detected by the RCA-qPCR method. **d** Serial dilutions of positive liver samples of HBV cccDNA were detected by the RCA-PCR-CRISPR method. **e** Serial dilutions of positive liver samples of HBV cccDNA were detected by ddPCR. Error bars indicate 1 S.D. based on three technical replicates; *p < 0.05, **p < 0.01, ***p < 0.001, ****p < 0.0001, compared with the no-input group

To further verify the effect of the detection method we established, we compared the qPCR, PCR-CRISPR, RCA-qPCR, RCA-PCR-CRISPR methods, and ddPCR and RCA-ddPCR detection as controls. Using serial dilutions of HBV cccDNA-positive liver samples, we discovered that the RCA-PCR-CRISPR achieved sensitivity to as little as one copy/μL of HBV cccDNA, which has similar levels to ddPCR and RCA-ddPCR, whereas PCR-CRISPR and RCA-qPCR could not detect when the HBV cccDNA concentration was less than ten copies/μl. Moreover, RCA-ddPCR detected ten times more copies of HBV cccDNA than ddPCR, but the sensitivity of the detection was the same (Fig. 5a–f). These results revealed that combining Cas13a-based detection with RCA and PCR amplification steps further increased the sensitivity to low levels of HBV cccDNA.

### Detection of HBV cccDNA in different kinds of clinical samples using qPCR, PCR-CRISPR, RCA-qPCR, RCA-PCR-CRISPR and ddPCR methods

**Fig. 6:**
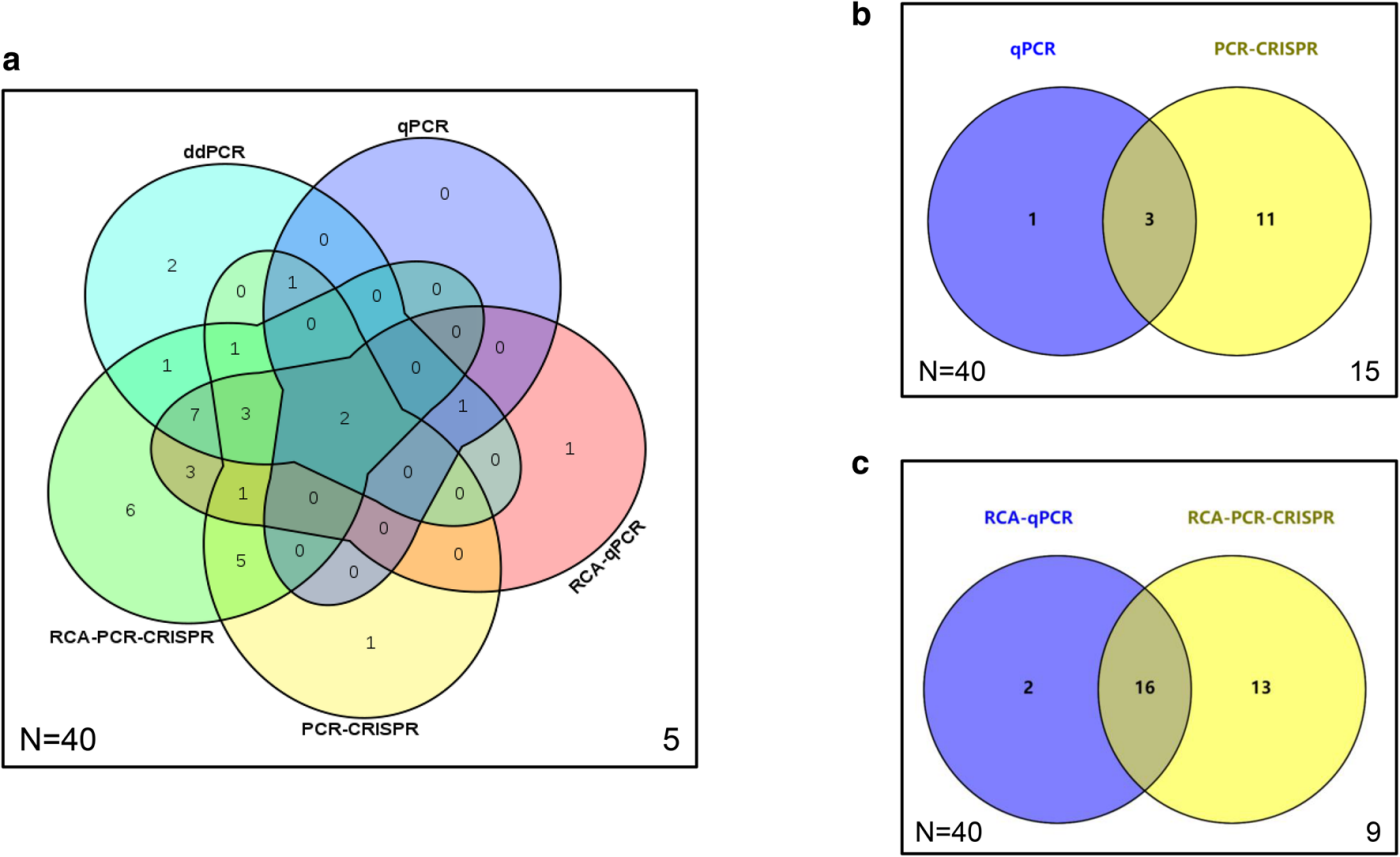
Venn diagrams comparing several detection methods for HBV cccDNA in liver tissue samples of clinical patients. **a** Venn diagrams comparing HBV cccDNA detection results of qPCR, PCR-CRISPR, RCA-qPCR, RCA-PCR-CRISPR and ddPCR for liver tissue of 40 clinical samples. **b** Venn diagrams comparing HBV cccDNA detection results of qPCR and PCR-CRISPR for liver tissue of 40 clinical samples. **c** Venn diagrams comparing HBV cccDNA detection results of RCA-qPCR and RCA-PCR-CRISPR for liver tissue of 40 clinical samples

We first tested HBV cccDNA in the liver tissues of 40 HBV-associated patients and 3 healthy individuals with the qPCR, PCR-CRISPR, RCA-qPCR, RCA-PCR-CRISPR and ddPCR methods. HBV DNA of all kinds of clinical samples was detected by the Abbott m2000 RealTime System in the Clinical Laboratory Center of Beijing YouAn Hospital, Capital Medical University (Supplementary Tables 1 and 2). HBV cccDNA was not detected by any method in the samples of 3 healthy people, while in 40 HBV-associated patients, 18 HBV cccDNA-positive samples were detected by ddPCR (positive rate: 45%, 18/40), 4 samples were detected by qPCR (positive rate: 10%, 4/40), 14 samples were detected by PCR-CRISPR (positive rate: 35%, 14/40), 18 samples were detected by RCA-qPCR (positive rate: 45%, 18/40), and 29 samples were detected by RCA-PCR-CRISPR (positive rate: 72.5%, 29/40) (Supplementary Table 1). The Venn diagram compares several detection methods and the Cas13a-based detection assay further enhanced the positive rate of HBV cccDNA in 40 liver tissue samples of clinical patients (Fig. 6a–c).

Then, we tested HBV cccDNA in plasma, whole blood and PBMC samples of 24 HBV-associated patients and 3 healthy individuals with qPCR, PCR-CRISPR, RCA-qPCR, and RCA-PCR-CRISPR methods. HBV cccDNA was detected by ddPCR in one plasma, three whole blood and one PBMC sample, by RCA-qPCR in two whole blood and one PBMC sample, and by RCA-PCR-CRISPR in two whole blood and one PBMC sample (Supplementary Table 2). The results suggested that the RCA-PCR-CRISPR has a high positive detection rate for HBV cccDNA; moreover, HBV cccDNA was rarely detected by these methods in the plasma, whole blood and PBMC samples of HBV-associated patients.

## Discussion

In this study, we developed a CRISPR-based detection method for HBV cccDNA by a method combining RCA and PCR called the CRISPR-based cccDNA assay. Compared with the existing HBV cccDNA detection methods, CRISPR-based cccDNA detection has theoretically significant sensitivity and specificity because it has the following characteristics: (1) the “collateral effect” of Cas13a further increases the amplified signals of RCA and PCR to improve the detection sensitivity, and (2) the crRNA design combine with Hind III digestion and PSAD-treated sample processing and cross-gap region primer design of PCR to further improve the specificity of detection. Therefore, the CRISPR-based cccDNA assay has enhanced sensitivity and specificity for detecting HBV cccDNA.

To improve the treatment of patients with HBV infection, we need better methods for detecting HBV cccDNA. In 2011, a study reported the use of nested qPCR to quantify cccDNA in PBMCs and bone marrow mononuclear cells (MMNCs); 42% of MMNC samples and 36% of PBMC samples tested positive for HBV cccDNA [18]. In this study, we developed a CRISPR-cccDNA assay with high sensitivity and specificity for HBV cccDNA detection. The advantage of this new method is the use of the CRISPR/Cas detection system, which involves a transcription step after PCR amplification for amplifying the RCA products. Moreover, when the target RNA is recognized, the collateral effect of Cas13a is triggered, resulting in additional signal amplification. For HBV cccDNA-positive samples, the sensitivity of the CRISPR-cccDNA assay was as low as 1 copy/μL, which was consistent with ddPCR and qPCR methods, while higher PCR-CRISPR and RCA-qPCR methods detected 10 copies/μL concentration of HBV cccDNA. Additionally, we tested the liver tissues of 40 HBV-associated patients. The results suggested that the positive coincidence rate of the CRISPR-cccDNA assay was higher than that of other methods. The purpose of current treatment is to achieve effective viral suppression, biochemical remission and histological improvement. Due to the existence of HBV cccDNA, no treatment can achieve a complete cure, and an effective method of detecting HBV cccDNA is therefore needed to assess the effect of treatment. Additionally, patients with HBV infection who have progressed to the later stages of the disease can be treated only with liver transplantation. The detection of HBV cccDNA can provide a better evaluation of the effect of liver transplantation. Consequently, our newly established CRISPR-based cccDNA assay to detect HBV cccDNA will help to evaluate clinical treatment effects and monitor relapse after treatment.

HBV cccDNA is well known to exist in HBV-infected liver cells. However, whether HBV cccDNA exists outside the liver has caused great controversy. Several reports have shown that HBV cccDNA was detected in plasma samples of patients and had a significant correlation with HBsAg titer [19]. Nevertheless, previous studies have suggested that HBV cccDNA does not exist in serum and cannot be formed in liver cells outside the liver [20]. There is no consensus. In this study, we tested the whole blood, PMBCs and plasma of 24 HBV-related patients using ddPCR, qPCR, RCA-qPCR, PCR-Cas13a and CRISPR-cccDNA assays. Our data revealed that HBV cccDNA was detected by ddPCR in one plasma, three whole blood and one PBMC sample, by RCA-qPCR in two whole blood and one PBMC sample, and by RCA-PCR-CRISPR in two whole blood and one PBMC sample. The RCA-PCR-CRISPR assay we established has high sensitivity and specificity, and HBV cccDNA was detected in a small number of blood samples with this method, which may be due to cell rupture and release into the blood. But too few samples were detected, and there was no correlation and statistical significance between these results, so we concluded that HBV cccDNA was not present in plasma, whole blood or PBMCs.

This study had several limitations. Although our CRISPR-cccDNA assay detects HBV cccDNA with high specificity and sensitivity, cccDNA quantification is not linear compared with qPCR and ddPCR (10^6^–10^1^ HBV DNA copies). We need to continue to explore the linearity of Cas13 detection. The approach involves two-step RCA amplification and PCR transcription, which are slightly complicated and need to be further optimized. Additionally, the liver tissues, plasma, whole blood and PBMCs of HBV-associated patients were detected by this novel method, which possesses high sensitivity for liver tissue detection. However, the plasma, whole blood and PBMC samples and liver tissue samples were not from the same patients, preventing the observation of a clear relationship between liver tissue and extrahepatic HBV cccDNA. Further experiments are required to investigate this issue.

## Conclusion

In summary, the CRISPR-based cccDNA assay is an ultrasensitive and highly specific approach for HBV cccDNA detection. It provides a powerful tool for clinical treatment and useful guidance for patients undergoing long-term anti-HBV therapy to improve their administration. Further study will be required to optimize the amplification detection steps of the method and verify the connection between liver tissue and extrahepatic HBV cccDNA.

## Supplementary Information

Below is the link to the electronic supplementary material.

Supplementary file1 (DOCX 56 KB)

## Data Availability

Not applicable.

## Abbreviations

HBV: Hepatitis B virus
cccDNA: Covalently closed circular DNA
PSAD: Plasmid-safe ATP-dependent DNase
RCA: Rolling circle amplification
PBMCs: Peripheral blood mononuclear cells
ddPCR: Droplet digital PCR
CHB: Cure of chronic hepatitis B
NAs: Nucleos(t)ide analogs
rcDNA: Relaxed circular DNA
qPCR: Quantitative real-time polymerase chain reaction
CRISPR-Cas: Clustered regularly interspaced short palindromic repeats-associated protein
dsDNA: Double-stranded DNA
ssDNA: Single-stranded DNA
crRNAs: CRISPR RNAs
HBsAg: HBV surface antigen
MMNCs: Marrow mononuclear cells
