# Supplementary material for: CRISPR/Cas13-assisted hepatitis B virus covalently closed circular DNA detection
Source: Hepatol Int. 2022 Mar 17;16(2):306–15. doi: 10.1007/s12072-022-10311-0 (PMC9013339; doi:10.1007/s12072-022-10311-0)
Supplement: Supplementary file 1 — Supplementary file1 (DOCX 56 KB) [file 12072_2022_10311_MOESM1_ESM.docx]

**Supplementary Methods:**

**DNA sample preparation and pretreatment**

Total DNA was extracted from liver tissue, whole blood, plasma and PBMCs using a QIAamp DNA Mini Kit (Qiagen, Germany) according to the manufacturer’s protocol. To increase the sensitivity and accuracy of cccDNA detection, total DNA was pretreated with HindIII restriction endonuclease (New England Biolabs, USA) according to the manufacturers’ instructions. HindIII has no restriction sites in cccDNA, and plasmid-safe ATP-dependent DNase (PSAD) (Lucigen, USA) was used to eliminate rcDNA, single-stranded DNA, and double-stranded DNA at 37°C for 12 hours and then 70°C for 30 minutes to stop the reaction. The total reaction volume was 11.2 μL, including 0.8 μL of ATP 25 mM, 2 μL of PSAD buffer, 0.4 μL of PSAD and 8.5 μl of total DNA.

**Amplification of HBV cccDNA**

Digested samples were used as templates for the next RCA using Phi29DNA polymerase (New England Biolabs, USA). The primers for RCA were R1: 5’-ACCTATTCTCCTCCC-3’ (nt 1758-1744), R2: 5’-ATGCAACTTTTTCAC-3’ (nt 1686-1700), R3: 5’-GGCCCACATATTGT-3’ (nt 2599-2585), R4: 5’-AATCCTCACAATACC-3’ (nt 99-113), R5: 5’-CTAGCAGAGCTTGGT-3’ (nt 29-15), R6:5’-CCTTTGTCCAAGGGC-3’ (nt 2689-2675), R7:5’-TAGAAGAAGAACTCC-3’ (nt 2240-2254), and R8:5’-CCTATGGGAGTGGGC-3’ (nt 510-524). The 10 µL of reaction mix included 1 µL of 10× reaction buffer, primers at a concentration of 10 µmol/L each, and 4 µL of PSAD-digested DNA, and the reaction was denatured at 95°C for 3 min followed by annealing at 50°C for 15 s, 30°C for 15 s and 20°C for 10 min and then placed on ice. The solution was then added to 20 mL of reaction mixture containing primers at a concentration of 10 mmol/L each, 1 µL of Phi29 DNA polymerase, 1 µL of 10× reaction buffer, 1 µL of Phi29 BSA, and 2.5 mM dNTPs. The reaction was carried out at 30°C for 16 h and terminated at 65°C for 10 min.

The 25-µL PCR mixture contained 12.5 µL 2×ExTaq Mix, 400 nM HBV cccDNA sense (5‘-GGGGCGCACCTCTCTTTA-3’, nt1523-1540) and HBV cccDNA antisense (5’-AGGCACAGCTTGGAGGC-3’, nt1886-1870) primers, and a 2 µL RCA DNA sample, which produced a 347-base pair amplicon. PCR amplification was carried out on a thermal cycler (Applied Biosystems, USA) with a thermal profile beginning at 94°C for 3 min, followed by 30 cycles at 94°C for 30 s, 58°C for 45 s, 1 cycle of 72°C for 10 min, and ending at 4°C.

**Quantification of HBV cccDNA by quantitative real-time PCR (qPCR)**

qPCR was performed using an ABI 7500 Fast DX (Applied Biosystems, Foster City, CA, USA) instrument. The forward and reverse primers were 5’- GGGGCGCACCTCTCTTTA-3’ and 5’-AGGCACAGCTTGGAGGC-3’, respectively, and the probe was 5’-FAM-TCACCTCTGCCTAATCATCTC-TAMRA-3’. A 20 µL qPCR reaction mix comprised 10 µL of Fast Advanced Master Mix (Thermo Fisher Scientific, Waltham, MA, USA), 10 nM primers and probe, 2 µL of RCA DNA template and 6.5 µL of deionized water. The amplification conditions were as follows: denaturation at 94°C for 3 min, followed by 40 cycles of denaturation at 94°C for 30 s and annealing at 58°C for 45 s.

**Quantification of HBV cccDNA by droplet digital PCR (ddPCR)**

ddPCR was performed on the TD-1^TM^ Droplet Digital^TM^ PCR system (TargetingOne, licensed in China, registration number: 20170025; 20190065; 20192220517) following the manufacturer’s instructions. Thirty microliters of reaction mix containing 7.5 μL of ddPCR Supermix (4×), 400 nM primers (used for qPCR), 200 nM probes (used for qPCR) and 15 μL of the RCA DNA template was added to the droplet generation chip. Then, 180 μL of droplet generation oil was added, and the chip was used for droplet generation by a Drop Maker (TargetingOne, Beijing China). The PCR conditions were as follows: 95°C predenaturation for 10 min, amplification for 40 cycles with denaturation at 95°C for 30 s, annealing at 60°C for 1 min, and cooling at 12°C for 5 min. After PCR, the amplification products were transferred into a droplet detection chip, which was loaded on a Chip Reader (TargetingOne, Beijing China) for fluorescence detection.

**Positive control of HBV cccDNA**

We used two approaches to create HBV cccDNA-positive templates. One method was to construct artificial cccDNA. A plasmid containing the partial HBV genome (GenBank ID: KM229703.1 site: 1501-1920) and pUC57 served as the templates to construct an artificial HBV cccDNA standard. Restriction endonuclease analysis was performed using BspQI (NEB), which has only one restriction site at two terminals of the 3.2 kb DNA fragment in the plasmid and then purified by agarose gel electrophoresis recovery, and 3.2 kb of DNA was connected by T4 ligase (NEB) to form a circularized genome as artificial cccDNA (Fig. 4A). Another method is to extract total DNA from the liver tissue of CHB patients and treat it with HindIII, PSAD and RCA, with a load of 10^5^ copies/μl quantified by ddPCR and as HBV cccDNA-positive samples.

**Preparation of CRISPR RNAs (crRNAs) for HBV cccDNA detection**

To prepare crRNAs, complete crRNA sequences were synthesized as single-stranded DNA and then PCR-amplified with an appended T7 promoter sequence. Two complementary pairs of crRNA DNA primers (final concentrations 10 μM) were annealed and incubated overnight with T7 polymerase at 37°C using the HiScribe T7 Quick High Yield RNA Synthesis Kit (New England Biolabs). crRNAs were then purified using an RNA Rapid Concentration Purification Kit (Sangon Biotech).

**CRISPR-Cas13a detection for HBV cccDNA**

Detection was conducted as follows: 1 µL of murine RNase inhibitor (New England Biolabs), 45 nM LwCas13a (Hangzhou ZC Bio-Sci &Tech Co., Ltd. in Hangzhou, China), 22.5 nM crRNA, 1 µL of NTP Mix, 0.4 μL of T7 RNA Polymerase, 2.5 µL of HELPS buffer solution, 125 nM quenched fluorescent RNA reporter (RNAse Alert, Thermo Scientific, Waltham, MA, USA), 5 µL of target nucleic acid and 10 μL of RNase-free water were mixed in nuclease assay buffer. Reactions were conducted at 37°C for 1 h and monitored on an ABI 7500 Fast DX (Applied Biosystems, Foster City, CA, USA) every 2 mins.

**Cell culture and transfection**

Huh7 cells were cultured in Dulbecco's modified Eagle medium (DMEM) (Gibco, Carlsbad, Calif, USA) with 10% fetal bovine serum (FBS) (Gibco, Carlsbad, Calif, USA), and were maintained in a humidified incubator at 37°C with 5% CO2. Lipofectamine 3000 (L3000015, Invitrogen, USA) was used to transfect plasmid. The procedure was carried out according to the instructions.

**Supplementary Table 1.** Results of clinical patient liver tissue sample

| **Sample**  **Number** | **HBV DNA (IU/mL)** | **HBV cccDNA** | | | | | |
| --- | --- | --- | --- | --- | --- | --- | --- |
|  |  | **ddPCR (copies/μL)** | **qPCR (copies/μL)** | **RCA-qPCR (copies/μL)** | **PCR- CRISPR** | **RCA-PCR-CRISPR** | |
| 1 | 7.28E+04 | 0 | 0 | 0 | - | | + |
| 2 | 1.37E+05 | 0 | 0 | 0 | + | | + |
| 3 | 6.41E+05 | 0 | 0 | 15441.83 | + | | + |
| 4 | 9.03E+06 | 332101.3 | 0 | 0 | + | | + |
| 5 | 5.65E+06 | 70.9 | 0 | 31271.34 | + | | + |
| 6 | 1.42E+06 | 427.9 | 0 | 31355.33 | + | | + |
| 7 | 4.36E+06 | 0 | 0 | 0 | + | | + |
| 8 | 3.75E+06 | 0 | 0 | 0 | - | | + |
| 9 | 1.2E+06 | 0 | 0 | 0 | - | | + |
| 10 | 4.98E+06 | 52.2 | 0 | 0 | - | | + |
| 11 | 2.2E+08 | 219832.1 | 10038.86 | 60315.13 | + | | + |
| 12 | 1.56E+05 | 0 | 0 | 0 | + | | + |
| 13 | 4.57E+06 | 509.3 | 0 | 53098.14 | - | | + |
| 14 | 7.41E+05 | 0 | 0 | 0 | - | | + |
| 15 | 8.42E+04 | 0 | 0 | 0 | - | | + |
| 16 | 2.26E+07 | 0 | 0 | 0 | - | | + |
| 17 | 1.42E+06 | 9.9 | 0 | 20604.3 | + | | + |
| 18 | 1.45E+07 | 0 | 0 | 0 | + | | + |
| 19 | 6.70E+06 | 0 | 0 | 0 | + | | - |
| 20 | 1.49E+05 | 0 | 0 | 0 | + | | + |
| 21 | 7.84E+05 | 0 | 0 | 81531.374 | - | | + |
| 22 | 3.32E+05 | 0 | 0 | 0 | - | | - |
| 23 | 3.73E+04 | 0 | 0 | 0 | - | | - |
| 24 | 6.43E+04 | 0 | 0 | 0 | - | | - |
| 25 | 1.55E+06 | 0 | 0 | 81027.425 | - | | + |
| 26 | 7.44E+05 | 6.3 | 0 | 204953.043 | - | | + |
| 27 | 4.72E+08 | 16.2 | 38018.940 | 3024516.705 | + | | + |
| 28 | 3.1E+06 | 2.1 | 0 | 166966.292 | - | | + |
| 29 | 8.8E+05 | 0 | 0 | 192016.770 | - | | + |
| 30 | 3.44E+06 | 0 | 0 | 0 | - | | - |
| 31 | 2.24E+05 | 0 | 0 | 0 | - | | - |
| 32 | 2.56E+06 | 0 | 0 | 21404.050 | - | | - |
| 33 | 1.72E+06 | 7.9 | 0 | 0 | - | | - |
| 34 | 2.74E+05 | 5.9 | 7943.282 | 19386.208 | - | | - |
| 35 | 7.94E+05 | 5.6 | 0 | 101825.747 | - | | + |
| 36 | 1.26E+06 | 34.7 | 0 | 53372.412 | - | | + |
| 37 | 1.63E+08 | 19 | 8301.384 | 0 | + | | - |
| 38 | 3.3E+05 | 4.7 | 0 | 564576.948 | - | | + |
| 39 | 2.52E+05 | 10.6 | 0 | 252671.813 | - | | + |
| 40 | 3.7E+05 | 24 | 0 | 0 | - | | - |
| 41 | 0 | 0 | 0 | 0 | - | | - |
| 42 | 0 | 0 | 0 | 0 | - | | - |
| 43 | 0 | 0 | 0 | 0 | - | | - |

**Supplementary Table2.** HBeAg/eAb status, HBsAg titer and ALT levels in 40 liver tissue samples of HBV-associated patients

| **Sample Number** | **HBV DNA (IU/mL)** | **HBeAg/eAb**  **status** | **HBsAg titer**  **(IU/mL)** | **ALT**  **(U/L)** |
| --- | --- | --- | --- | --- |
| 1 | 7.28E+04 | -/+ | 782 | 22 |
| 2 | 1.37E+05 | +/- | 1595 | 93 |
| 3 | 6.41E+05 | -/+ | 174 | 59 |
| 4 | 9.03E+06 | -/+ | 2113 | 45 |
| 5 | 5.65E+06 | +/- | 3029 | 34 |
| 6 | 1.42E+06 | -/+ | 3197 | 71 |
| 7 | 4.36E+06 | +/- | 6203 | 117 |
| 8 | 3.75E+06 | -/+ | 1021 | 98 |
| 9 | 1.2E+06 | +/- | 1765 | 74 |
| 10 | 4.98E+06 | -/+ | 1060 | 71 |
| 11 | 2.2E+08 | +/- | 13624 | 23 |
| 12 | 1.56E+05 | +/- | 6560 | 17 |
| 13 | 4.57E+06 | +/- | 1233 | 64 |
| 14 | 7.41E+05 | +/- | 5773 | 31 |
| 15 | 8.42E+04 | +/- | 363 | 10 |
| 16 | 2.26E+07 | +/- | 24857 | 25 |
| 17 | 1.42E+06 | +/- | 2573 | 40 |
| 18 | 1.45E+07 | +/- | 17359 | 62 |
| 19 | 6.70E+06 | +/- | 5030 | 68 |
| 20 | 1.49E+05 | +/- | 3499 | 32 |
| 21 | 7.84E+05 | +/- | 873 | 51 |
| 22 | 3.32E+05 | +/- | 3874 | 67 |
| 23 | 3.73E+04 | -/+ | 2940 | 70 |
| 24 | 6.43E+04 | +/- | 486 | 17 |
| 25 | 1.55E+06 | +/- | 4791 | 35 |
| 26 | 7.44E+05 | +/- | 3542 | 23 |
| 27 | 4.72E+08 | +/- | 42851 | 35 |
| 28 | 3.1E+06 | +/- | 5701 | 25 |
| 29 | 8.8E+05 | -/+ | 4534 | 54 |
| 30 | 3.44E+06 | +/- | 2162 | 84 |
| 31 | 2.24E+05 | +/- | 3712 | 26 |
| 32 | 2.56E+06 | +/- | 2853 | 31 |
| 33 | 1.72E+06 | -/+ | 6351 | 19 |
| 34 | 2.74E+05 | -/+ | 3274 | 24 |
| 35 | 7.94E+05 | +/- | 2970 | 47 |
| 36 | 1.26E+06 | -/+ | 5248 | 36 |
| 37 | 1.63E+08 | +/- | 60732 | 62 |
| 38 | 3.3E+05 | +/- | 3751 | 28 |
| 39 | 2.52E+05 | +/- | 4942 | 21 |
| 40 | 3.7E+05 | +/- | 869 | 42 |

**Supplementary Table 3.** Results of clinical patient blood sample

| **Sample**  **Number** | **HBV DNA Viral load (IU/mL)** | **Types of samples** | **HBV cccDNA** | | | | |
| --- | --- | --- | --- | --- | --- | --- | --- |
|  |  |  | **ddPCR** **(copies/uL)** | **qPCR**  **(copies/uL)** | **RCA-qPCR**  **(copies/uL)** | **PCR- CRISPR** | **RCA-PCR-CRISPR** |
| 1 | 5.6×10^3^ | Plasma | 0 | 0 | 0 | - | - |
|  |  | Whole blood | 11.3 | 0 | 0 | - | - |
|  |  | PBMC | 0 | 0 | 0 | - | - |
| 2 | 2.7×10^4^ | Plasma | 0 | 0 | 0 | - | - |
|  |  | Whole blood | 12.8 | 0 | 0 | - | - |
|  |  | PBMC | 0 | 0 | 0 | - | - |
| 3 | 2.02×10^5^ | Plasma | 0 | 0 | 0 | - | - |
|  |  | Whole blood | 0 | 0 | 2.41×10^4^ | - | + |
|  |  | PBMC | 0 | 0 | 0 | - | - |
| 4 | 4.12×10^6^ | Plasma | 0 | 0 | 0 | - | - |
|  |  | Whole blood | 0 | 0 | 0 | - | - |
|  |  | PBMC | 0 | 0 | 0 | - | - |
| 5 | 5.5×10^7^ | Plasma | 0 | 0 | 0 | - | - |
|  |  | Whole blood | 34.8 | 0 | 8.04×10^7^ | - | + |
|  |  | PBMC | 0 | 0 | 0 | - | - |
| 6 | 1.4×10^8^ | Plasma | 0 | 0 | 0 | - | - |
|  |  | Whole blood | 0 | 0 | 0 | - | - |
|  |  | PBMC | 0 | 0 | 0 | - | - |
| 7 | 10^3^ | Plasma | 0 | 0 | 0 | - | - |
|  |  | Whole blood | 0 | 0 | 0 | - | - |
|  |  | PBMC | 0 | 0 | 0 | - | - |
| 8 | 10^3^ | Plasma | 0 | 0 | 0 | - | - |
|  |  | Whole blood | 0 | 0 | 0 | - | - |
|  |  | PBMC | 0 | 0 | 0 | - | - |
| 9 | 10^5^ | Plasma | 0 | 0 | 0 | - | - |
|  |  | Whole blood | 0 | 0 | 0 | - | - |
|  |  | PBMC | 12.5 | 0 | 0 | - | - |
| 10 | 10^6^ | Plasma | 0 | 0 | 0 | - | - |
|  |  | Whole blood | 0 | 0 | 0 | - | - |
|  |  | PBMC | 0 | 0 | 0 | - | - |
| 11 | 10^3^ | Plasma | 0 | 0 | 0 | - | - |
|  |  | Whole blood | 0 | 0 | 0 | - | - |
|  |  | PBMC | 0 | 0 | 0 | - | - |
| 12 | 10^4^ | Plasma | 0 | 0 | 0 | - | - |
|  |  | Whole blood | 0 | 0 | 0 | - | - |
|  |  | PBMC | 0 | 0 | 0 | - | - |
| 13 | 10^5^ | Plasma | 3.8 | 0 | 0 | - | - |
|  |  | Whole blood | 0 | 0 | 0 | - | - |
|  |  | PBMC | 0 | 0 | 0 | - | - |
| 14 | 10^6^ | Plasma | 0 | 0 | 0 | - | - |
|  |  | Whole blood | 0 | 0 | 0 | - | - |
|  |  | PBMC | 0 | 0 | 0 | - | - |
| 15 | 10^7^ | Plasma | 0 | 0 | 0 | - | - |
|  |  | Whole blood | 0 | 0 | 0 | - | - |
|  |  | PBMC | 0 | 0 | 0 | - | - |
| 16 | 10^8^ | Plasma | 0 | 0 | 0 | - | - |
|  |  | Whole blood | 0 | 0 | 0 | - | - |
|  |  | PBMC | 0 | 0 | 0 | - | - |
| 17 | 10^5^ | Plasma | 0 | 0 | 0 | - | - |
|  |  | Whole blood | 0 | 0 | 0 | - | - |
|  |  | PBMC | 0 | 0 | 0 | - | - |
| 18 | 10^6^ | Plasma | 0 | 0 | 0 | - | - |
|  |  | Whole blood | 0 | 0 | 0 | - | - |
|  |  | PBMC | 0 | 0 | 0 | - | - |
| 19 | 10^6^ | Plasma | 0 | 0 | 0 | - | - |
|  |  | Whole blood | 0 | 0 | 0 | - | - |
|  |  | PBMC | 0 | 0 | 0 | - | - |
| 20 | 10^8^ | Plasma | 0 | 0 | 0 | - | - |
|  |  | Whole blood | 0 | 0 | 0 | - | - |
|  |  | PBMC | 0 | 0 | 0 | - | - |
| 21 | 3179 | Plasma | 0 | 0 | 0 | - | - |
|  |  | Whole blood | 0 | 0 | 0 | - | - |
|  |  | PBMC | 0 | 0 | 0 | - | - |
| 22 | 194 | Plasma | 0 | 0 | 0 | - | - |
|  |  | Whole blood | 0 | 0 | 0 | - | - |
|  |  | PBMC | 0 | 0 | 0 | - | - |
| 23 | 7.61×10^5^ | Plasma | 0 | 0 | 0 | - | - |
|  |  | Whole blood | 0 | 0 | 0 | - | - |
|  |  | PBMC | 0 | 0 | 0 | - | - |
| 24 | 2.61×10^5^ | Plasma | 0 | 0 | 0 | - | - |
|  |  | Whole blood | 0 | 0 | 0 | - | - |
|  |  | PBMC | 0 | 0 | 7.92×10^3^ | - | + |
| 26 | 0 | Plasma | 0 | 0 | 0 | - | - |
|  |  | Whole blood | 0 | 0 | 0 | - | - |
|  |  | PBMC | 0 | 0 | 0 | - | - |
|  |  | Plasma | 0 | 0 | 0 | - | - |
| 27 | 0 | Whole blood | 0 | 0 | 0 | - | - |
|  |  | PBMC | 0 | 0 | 0 | - | - |
|  |  | Plasma | 0 | 0 | 0 | - | - |
| 28 | 0 | Whole blood | 0 | 0 | 0 | - | - |
|  |  | PBMC | 0 | 0 | 0 | - | - |

**Supplementary Table4.** HBeAg/eAb status, HBsAg titer and ALT levels in 24 blood samples of HBV-associated patients

| **Sample Number** | **HBV DNA (IU/mL)** | **HBeAg/eAb**  **status** | **HBsAg titer**  **(IU/mL)** | **ALT**  **(U/L)** |
| --- | --- | --- | --- | --- |
| 1 | 5.6×10^3^ | +/- | 7306 | 47 |
| 2 | 2.7×10^4^ | -/+ | 436 | 32 |
| 3 | 2.02×10^5^ | -/+ | 1401 | 46 |
| 4 | 4.12×10^6^ | +/- | 7570 | 29 |
| 5 | 5.5×10^7^ | +/- | 5563 | 44 |
| 6 | 1.4×10^8^ | +/- | 60452 | 14 |
| 7 | 10^3^ | +/- | 4892 | 15 |
| 8 | 10^3^ | -/+ | 3398 | 20 |
| 9 | 10^5^ | +/- | 2094 | 31 |
| 10 | 10^6^ | -/+ | 2990 | 28 |
| 11 | 10^3^ | +/- | 3771 | 22 |
| 12 | 10^4^ | +/- | 3462 | 51 |
| 13 | 10^5^ | +/- | 6385 | 35 |
| 14 | 10^6^ | +/- | 5379 | 24 |
| 15 | 10^7^ | +/- | 8634 | 57 |
| 16 | 10^8^ | +/- | 35355 | 30 |
| 17 | 10^5^ | -/+ | 4683 | 16 |
| 18 | 10^6^ | +/- | 5612 | 39 |
| 19 | 10^6^ | +/- | 2263 | 34 |
| 20 | 10^8^ | +/- | 11700 | 23 |
| 21 | 3179 | -/+ | 1123 | 19 |
| 22 | 194 | +/- | 635 | 25 |
| 23 | 7.61×10^5^ | +/- | 7365 | 22 |
| 24 | 2.61×10^5^ | +/- | 4151 | 17 |

**Supplementary Table5.** Sequences and positions of HBV cccDNA qPCR primers.

| name | Sequences | positions |
| --- | --- | --- |
| sense | 5’-GGGGCGCACCTCTCTTTA-3’ | 1523-1540 |
| antisense | 5’-AGGCACAGCTTGGAGGC-3’ | 1886-1870 |
| probe | 5’-FAM-TCACCTCTGCCTAATCATCTC-TAMRA-3’ | 1825-1845 |

**Supplementary Table6.** Sequences and positions of HBV cccDNA RCA primers.

| name | Sequences | positions |
| --- | --- | --- |
| R1 | 5’-ACCTATTCTCCTCCC-3’ | 1758-1744 |
| R2 | 5’-ATGCAACTTTTTCAC-3’ | 1686-1700 |
| R3 | 5’-GGCCCACATATTGT-3’ | 2599-2585 |
| R4 | 5’-AATCCTCACAATACC-3’ | 99-113 |
| R5 | 5’-CTAGCAGAGCTTGGT-3’ | 29-15 |
| R6 | 5’-CCTTTGTCCAAGGGC-3’ | 2689-2675 |
| R7 | 5’-TAGAAGAAGAACTCC-3’ | 2240-2254 |
| R8 | 5’-CCTATGGGAGTGGGC-3’ | 510-524 |

**Supplementary Table7.** CRISPR/Cas13a-related sequences used in this study. T7 promoter sequences are colored in red and spacer sequences in crRNA are colored in blue.

| name | Sequences |
| --- | --- |
| HBV cccDNA-F | AATTCTAATACGACTCACTATAGGG GGGGCGCACCTCTCTTTA |
| HBV cccDNA-R | AGGCACAGCTTGGAGGC |
| HBV cccDNA crRNA1 | GGGGAUUUAGACUACCCCAAAAACGAAGGGGACUAAAAC UCACCUCUGCCUAAUCAUCUCUUGUUCA |
| HBV cccDNA crRNA2 | GGGGAUUUAGACUACCCCAAAAACGAAGGGGACUAAAAC AACUUUUUCACCUCUGCCUAAUCAUCUC |
| HBV cccDNA crRNA3 | GGGGAUUUAGACUACCCCAAAAACGAAGGGGACUAAAAC UUUUCACCUCUGCCUAAUCAUCUCUUGU |
